# Rapid visual characterization of alkaloid changes in traditional processing of Tibetan medicine *Aconitum pendulum* by high-performance thin-layer chromatography coupled with desorption electrospray ionization mass spectrometry imaging

**DOI:** 10.3389/fphar.2023.1104473

**Published:** 2023-04-21

**Authors:** Xiaoyan Tan, Qingxiu He, Zhaoqing Pei, Yue Liu, Zige Feng, Congying Li, Ce Tang, Yi Zhang

**Affiliations:** ^1^ School of Pharmacy, Chengdu University of Traditional Chinese Medicine, Chengdu, China; ^2^ School of Ethnic Medicine, Chengdu University of Traditional Chinese Medicine, Chengdu, China; ^3^ Innovative Institute of Chinese Medicine and Pharmacy, Chengdu University of Traditional Chinese Medicine, Chengdu, China; ^4^ Meishan Hosptial of Chengdu University of Traditional Chinese Medicine, Meishan, China

**Keywords:** DESI-MSI, HPTLC, radix aconiti, processing, alkaloids

## Abstract

Radix Aconiti, also known as Tie-bang-chui (TBC), Pang-a-na-bao, and Bang-na, is a typical aconitum Tibetan medicine and a perennial herb of the genus *Aconitum pendulum* Busch. and *A. flavum* Hand. -Mazz. dry roots. It has high toxicity and remarkable efficacy; as such, it is a typical “highly toxic and effective” drug that needs be processed and used. Processing methods of this Tibetan medicine include non-heating of highland barley wine (HBW) and fructus chebulae soup (FCS). This work aimed to understand differences in chemical composition between non-heat processed products and raw TBC. In this study, high-performance thin-layer chromatography (HPTLC) and desorption electrospray ionization mass spectrometry imaging (DESI-MSI) were used to analyze the chemical composition of TBC processed by FCS (F-TBC) and HBW (H-TBC). The MRM mode of HPLC-QqQ-MS/MS was selected to determine the changes of several representative alkaloids to comparison with the former results. A total of 52 chemical constituents were identified in raw and processed products, and the chemical composition of F-TBC and H-TBC changed slightly compared with that of raw TBC. The processing mechanism of H-TBC was also different from that of F-TBC, which might be related to the large amount of acidic tannins in FCS. It was found that the content of all six alkaloids decreased after processing by FCS, and all five alkaloids decreased except aconitine increased after processing by HBW. The combination of HPTLC and DESI-MSI could be an effective method for rapid identification of chemical components and changing rules in ethnic medicine. The wide application of this technology provides not only an alternative method for the traditional separation and identification of secondary metabolism but also a reference for research on the processing mechanism and quality control of ethnic medicine.

## 1 Introduction

Radix Aconiti, also known as Tie-bang-chui (TBC), Pang-a-na-bao, Bang-na in Tibetan medicine, is a perennial herb of the genus *Aconitum pendulum* Busch. and *A. flavum* Hand. -Mazz. dry root and is mainly distributed in Qinghai, Gansu, Sichuan, Tibet and other regions of China. (Wang et al., 2016). TBC is a traditional medicinal material used in Tibetan medicine; which is sweet, slightly bitter, heat, and highly toxic and has remarkable efficacy. It is a typical “highly toxic and effective” drug with both strong toxicity and excellent efficacy. TBC is used to expel cold and relieve pain, dispel wind, and calm shock mainly for *Long* disease, cold disease, *Huangshui* disease, leprosy, madness, and so on (Dimaer, 1986).

**FIGURE 1:**
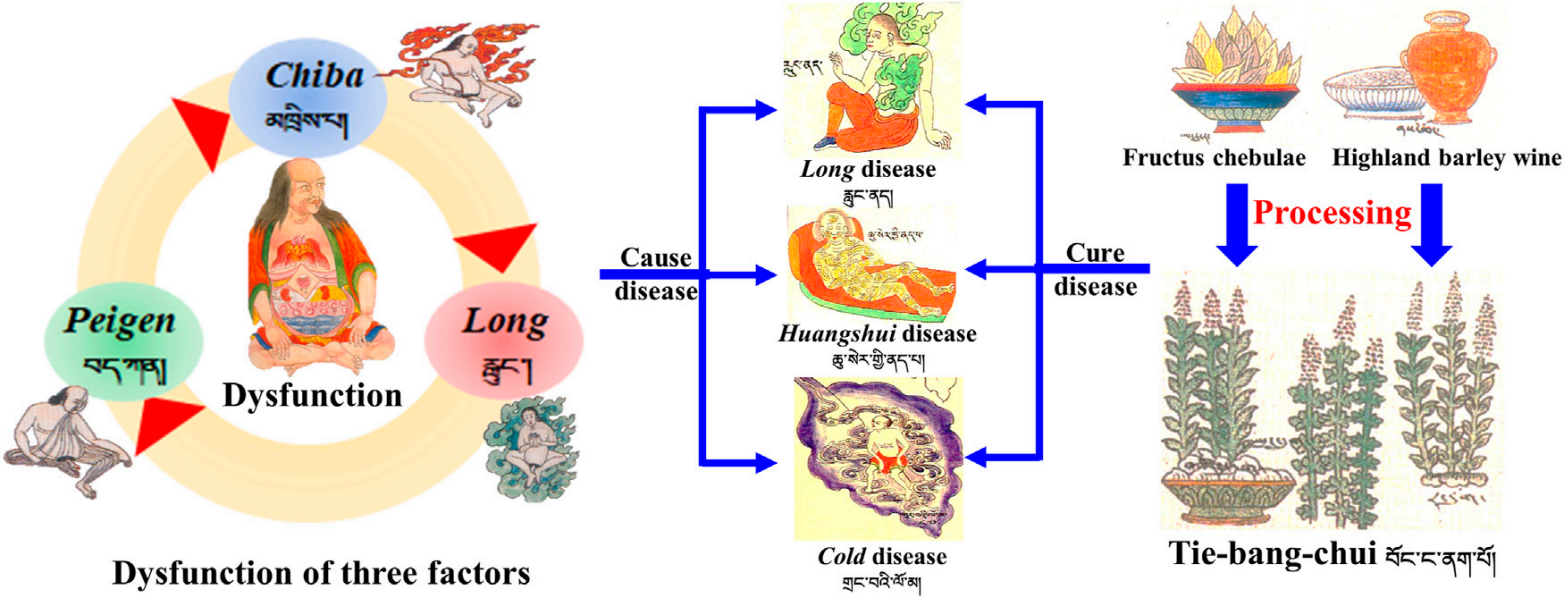
Traditional processing method of TBC in Tibetan medicine.

TBC is commonly used as a highly poisonous aconite Tibetan medicine. Many processing methods are used for TBC in ancient and modern times to ensure its safety; these methods include heat processing (baking, stir-frying, simmering, steaming, boiling, etc.) and non-heat processing (liquid auxiliary material soaking, bleaching, etc.) (Li et al., 2022). The main materials used for processing this Tibetan medicine are highland barley wine (HBW) and fructus chebulae soup (FCS) (Figure 1). Previous literature review and on-the-spot investigation showed that non-heat processing methods are often used in Tibetan hospitals and medicine factories. However, research on the related mechanism of non-heat processing is still in the primary stage and needs further work.

Various analytical methods, such as thin layer chromatography (TLC), high-performance liquid chromatography (HPLC), liquid chromatography mass spectrometry (LC-MS), and so on, have been used to determine aconitum alkaloid components. HPLC separation is the most commonly used method, but it has some disadvantages, such as long analysis time, complex preparation of mobile phase, and high solvent consumption (Csupor et al., 2007; Zhi et al., 2020). Therefore, a time-saving method with low reagent requirement should be developed to visualize changes in alkaloid composition of non-heat processed TBC.

Desorption electrospray ionization (DESI) was first proposed by Cooks team. DESI has rapid, highly sensitive *in situ* detection and has made great progress, especially *in situ* tumor analysis, *in situ* brain imaging, real-time dynamic change detection of chemical composition in the processing of traditional Chinese medicine, etc. This method has been widely used in food, drugs, forensic medicine, environment, and other fields (Milojković-Opsenica et al., 2022).

TLC detection is the simplest and most effective method for central control of almost all chemical drugs and quality control of traditional Chinese medicines and their excipients, which was used for quality control analysis in nearly 94% of the varieties in the first part of the Chinese Pharmacopoeia in the 2020 edition and nearly 40 varieties in the fourth part of the Chinese Pharmacopoeia in the fourth part of the excipients. Low cost, flexible and convenient operation, simple sample pre-treatment, high throughput and easy instrument coupling are the outstanding advantages of TLC. High-performance thin-layer chromatography (HPTLC) is a thin-layer chromatographic method using a higher separation efficiency thin-layer plate, which has improved separation, sensitivity and reproducibility compared to conventional TLC. HPTLC is commonly used in drug analysis but is often insufficient to obtain information on chemical composition. HPTLC involves chromatography for separation and can be combined with DESI-MSI to obtain better identification ability than DESI-MSI or HPTLC alone. In recent years, HPTLC-DESI-MSI has been used to analyze plant metabolites and peptides (Bagatela et al., 2015; Kaddi et al., 2015; Liu et al., 2022). Therefore, HPTLC has been proved to be a simple, rapid, efficient, and solvent-saving technique for the separation and characterization of chemical components in complex samples. However, no research has investigated the use of HPTLC-DESI-MSI to separate and detect dynamic changes in chemical composition in the processing of ethnic medicines.

In this study, TBC samples from unheated FCS and HBW were prepared. TBC samples and mixed reference substances were separated on HPTLC and detected by DESI-MSI. According to the constructed database and reference materials, the chemical components of different processed products were qualitatively detected. Changes in alkaloid content in non-heat processed TBC were characterized quickly and intuitively. In order to verify the feasibility of using HPTLC-DESI-MSI to rapidly monitor the changes of the constituents before and after processing, the MRM mode of HPLC-QqQ-MS/MS was selected to determine the changes of several representative alkaloids to comparison with the former results, and it was found that the trends of the changes were consistent, which proved the feasibility of the method.

## 2 Materials and methods

### 2.1 Materials and reagents

The reference standards (HPLC>98%) of aconitine (39), acetylaconitine (42), mesaconitine (37), deoxyaconitine (36), hypaconitine (33), and benzoylaconine (31) were purchased from PUSH Biotechnology Co., Ltd. (Chengdu, China). Reserpine (DST Biological Co., Ltd., HPLC≥98%, Lot No. DST210628-056). LC-MS-grade methanol, acetonitrile and formic acid were obtained from Sigma–Aldrich (United States). HPTLC silica 60 F_254_ was acquired from Merck KGaA (Darmstadt, Germany). Analytical-grade anhydrous ether, chloroform, 25% ammonia, and methanol were provided by Chengdu Kelong Chemical Co., Ltd. (Chengdu, China). HBW was produced by Qinghai Huzhu Highland Barley Wine Co., Ltd. (Huzhu, China). UPLC-grade ultrapure water was produced by Elga Labwater Purelab system (Elga-Veolia, High Wycombe, United Kingdom). Leucine encephalin (LE) as internal standard (IS) was supplied by Waters (Waters Corporation, United States). TBC samples were collected from Qinghai. FCS was purchased from Chengdu New Lotus Market and identified by Professor Zhang Yi of Chengdu University of Traditional Chinese Medicine. The related specimens were stored in the School of Ethnic Medicine, Chengdu University of Traditional Chinese Medicine.

### 2.2 Instruments

Lac part analytical weighing scales (Shanghai Liangping Instrumentation Co.), Ultrasonic Machine (Ningbo New Yicai Ultrasonic Equipment Co., Ltd.), Rotary evaporator (BUCHI Rotavapor R-300, Switzerland), Nitrogen blowing instrument (KL-512), Waters Snapt Q-Tof mass spectrometer equipped with a DESI source (United States), High performance liquid chromatograph (Model 1260) Tandem triple quadrupole mass spectrometer (Model 6420) (Agilent, United States), ShimNex CS HPLC C_18_ column (5μm, 4.6 mm × 250 mm).

### 2.3 Sample preparation

FCS were obtained by boiling FC 50 g with 1500 mL of water for 30 min, filtered to obtain filtrate, repeat boiling three times, consolidated filtrate, concentrated to 500 mL by rotary evaporator and cooled.

For traditional Tibetan medicine processing of TBC (Li et al., 2022), an appropriate amount of TBC powder was placed in a mortar, added three times the amount of HBW, soaked for 1 h, ground for 5 h, fermented at room temperature for 1 day, and dried to obtain TBC processed by HBW (H-TBC). TBC and three times amounts of FCS were obtained for 2 days, turned every 4 h, soaked, and dried to obtain TBC processed by FCS (F-TBC).

In brief, 2.5 g of powders of raw TBC (R-TBC), H-TBC, and F-TBC were accurately weighed and placed in 100 mL stopper conical flask. The mixture was added by 35 mL of the mixed solution of ether trichloromethane (3/1, *v*/*v*) and 3 mL of ammonia test solution. The mixture was shaken, extracted by ultrasound for 1 h, stood for 10 min, and filtered. The residue was discarded and concentrated by nitrogen blowing. The residue was transferred to a 25 mL volumetric flask with acetonitrile for constant volume (Lin et al., 2011). The sample solution was diluted to the appropriate concentration before use, and then filtered through a 0.22 µm filter membrane before testing.

Appropriate amounts of aconitine, acetyl aconitine, hypoaconitine, mesaconitine, deoxyaconitine, and benzoyl aconitine were accurately weighed. The mixed reference solution (D-TBC) containing 1.7 mg of aconitine (39), 1.50 mg of acetyl aconitine (42), 1.9 mg of hypoaconitine (33), 1.1 mg of mesaconitine (37), 1.6 mg of deoxyaconitine (36), and 1.4 mg of benzoyl aconitine (31) was prepared by adding acetonitrile and shaking well. The internal standard solution was prepared by weighing 1 mg of reserpine, dissolving it in acetonitrile, transferring it to a 50 mL volumetric flask, fixing the volume with acetonitrile, and filtering it through a 0.22 μm microporous organic membrane, waiting to be used.

### 2.4 HPTLC separation

Silica gel-precoated HPTLC plate (200 mm × 100 mm × 0.2 mm) was used for HPTLC separation. The extract solution or D-TBC with a volume of 5 µL was continuously sampled onto the HPTLC plate with a quantitative capillary. Cyclohexane/ethyl acetate/diethylamine (8/2/1, *v*/*v*/*v*) was used as the mobile phase. The sample was presaturated at room temperature (25°C) for 20 min and unfolded in the mobile phase until the front of the solvent reached the defined finish line. Finally, the sample was taken out, dried naturally, and detected by mass spectrometry.

### 2.5 HPTLC-DESI-MSI detection

**FIGURE 2:**
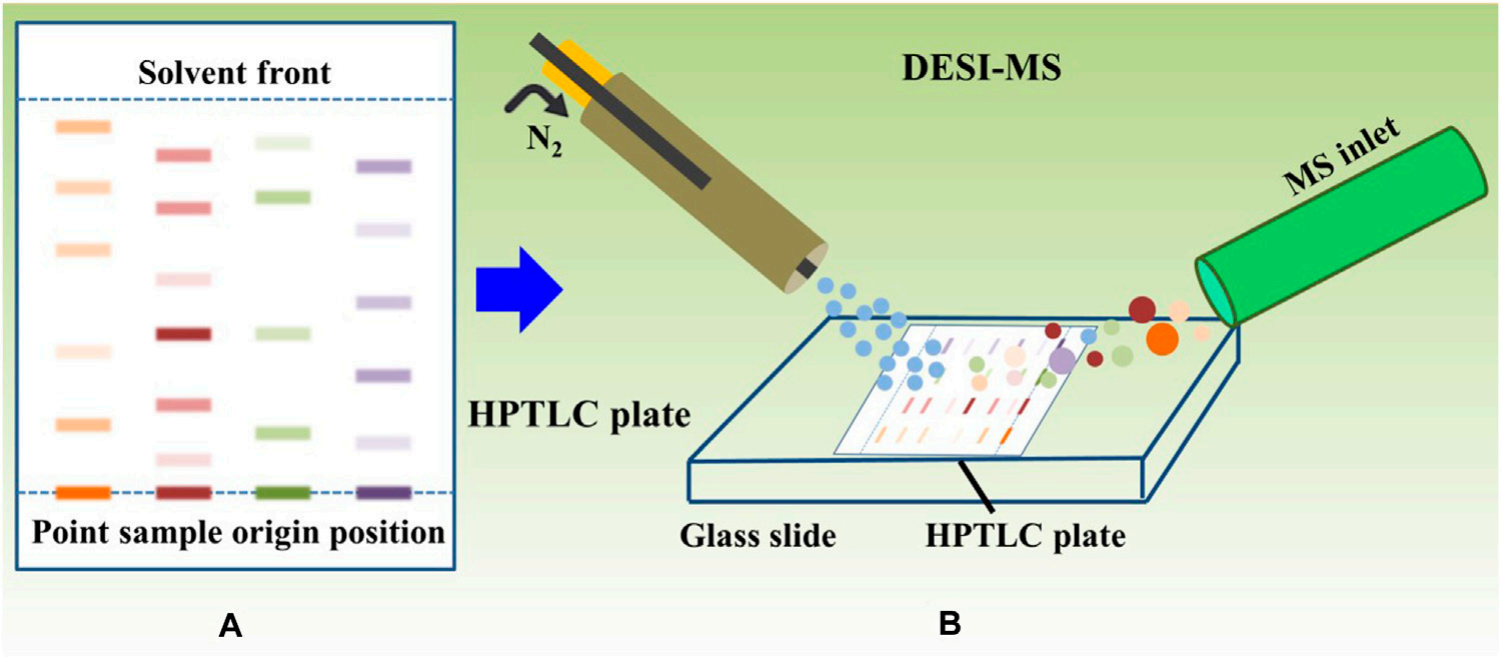
Detection schematic diagram of HPTLC-DESI-MSI testing process.

DESI-MSI experiment was carried out on a Waters Synapt G2-SI Q-TOF mass spectrometer equipped with a DESI source (Figure 2). The DESI parameters were optimized to obtain good ion signal intensity: nebulizing gas (nitrogen) pressure of 0.45 MPa; spray solvent of 70% methanol water, 30% H_2_O, 0.2% formic acid, and 0.1 mM LE at a flow rate of 2 μL min^-1^; capillary voltage of 4.5 kV; and positive ionization mode. The pixel size (150 μm X and Y pixel size) was determined based on the total scanning time of the mass spectrogram and the speed of the X–Y pixel size scanner. The mass range was m/z 100–1000, and the scanning speed was 300 μm s^-1^. HDI software was used to process raw MS files and create and view MSI.

### 2.6 HPTLC-DESI-MSI data processing

The MS raw data file was imported into HDI for imaging, and the regions of interests (ROIs) expanded by four points were exported. The self-built database, retention factor (Rf), and reference materials were used for chemical composition identification.

### 2.7 HPLC -QqQ-MS detection

#### 2.7.1 HPLC-QqQ-MS data acquisition

The mobile phase consisted of 0.1% formic acid water (A) and pure acetonitrile (B), the flow rate was 0.4  mL min^−1^, column temperature was maintained at 25°C, sample injection volume was 3 μL, and the gradient elution were as follows: 0–10 min, 23%–25% B; 10–25 min, 25%–45% B; 25–35 min, 45%–60% B; 35–45 min, 60%–60% B; 45–50 min, 60%–100%. The negative and positive ion modes were compared for the MS analysis. The positive mode resulted in a higher sensitivity and cleaner mass spectral background than the negative mode. ESI ion source, positive ion mode detection, atomization temperature: 300°C, nitrogen flow rate 5 L min^-1^, nebulizing gas pressure: 45 psi, capillary voltage: 3500 V. The collision energy and fragment ions voltage parameters were optimized as follows Table 1.

**TABLE 1:** Mass spectrum parameter information of each reference substance.

Compound	T_R_/min	Precursor ion *m/z*	Product ion *m/z*	F(V)	CE
Benzoylaconine	19.42	604.4	105.1	190	48
Mesaconitine	36.35	632.5	572.4	150	42
Aconitine	37.34	646.5	586.4	200	40
Acetylaconitine	37.53	688.3	628.2	200	42
Hypaconitine	37.36	616.5	524.2	150	43
Deoxyaconitine	38.14	630.2	570.2	200	41
Reserpine	37.95	609.1	194.9	200	30

#### 2.7.2 Method validation

**FIGURE 3:**
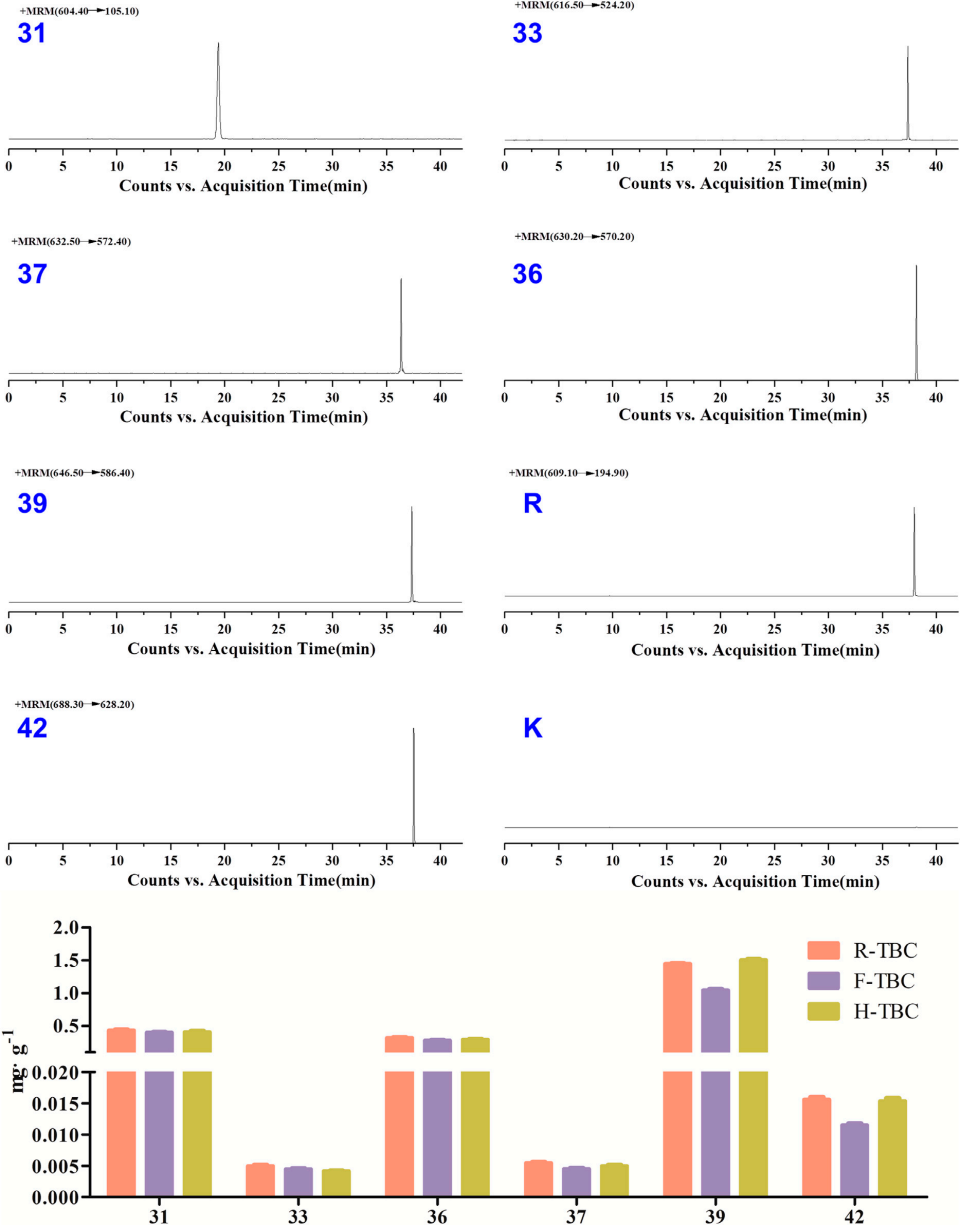
HPLC–QqQ–MS EIC of the 6 reference standard compounds, internal standard (reserpine, R) and blank solvent (B).

The specificity of the method was inspected by ion flow diagrams of the blank solvent and different channels for each control alkaloid (Figure 3 and Supplementary Table S1). From the ion flowdiagram, it can be seen that the method has good specificity.

To measure the sensitivity and precision of the method based on MRM mode of HPLC-QqQ-MS/MS, the standard curve, linear range, recovery, limit of detection (LOD), limit of quantification (LOQ) and precision were examined. The precision was measured by calculating the relative standard deviation (RSD) of the intra- and inter-day variations in the signal intensity (peak area). Recoveries were determined using R-TBC sample spiked with standard alkaloids. The recovery was calculated with the following equation: recovery (%) = (actual test content - found content)/spiked content *100%. LOD and LOQ were evaluated at signal-to-noise ratios (S/N) of 3:1 and 10:1, respectively. Each standard of alkaloids was weighed and dissolved in acetonitrile to get a concentration about of 300  μg mL^-1^. A standard mixture was obtained by mixing the individual alkaloids standard solution, and then diluted to yield concentration ranges of benzoylaconine, mesaconitine, aconitine, acetylaconitine, hypaconitine, deoxyaconitine are 1.11–355.84, 0.09–143.36, 1.34–855.04, 0.15–240.64, 0.12–38.4 and 0.13–165.89 ng mL^-1^ separately for the construction of standard curves. For the intra-day variability test, the samples were analyzed six times within 1 day; while for the inter-day variability test, the samples were examined on three consecutive days. Three repetitions were analyzed.

Standard curves for six different concentrations of alkaloids were constructed by plotting the signal intensity (ratio of compound to internal standard peak area) versus concentration in HPLC-QqQ-MS/MS analysis. The standard curves, linearity, correlation, linear range, LOD, LOQ, intra-day and inter-day precision and stability are detailed in Table 2. The recovery rates of the six alkaloids are shown in Table 3. The standard curves of the six alkaloids showed good linear correlation (*R* ^2^ > 0.999) in a certain concentration range. The values of LOD and LOQ obtained in HPLC-QqQ-MS/MS assay are less than 0.01 and 0.02 ng mL^-1^. The intra-day and the inter-day RSD of signal intensity (peak area) were less than 2.73% and 2.82%, respectively. Besides, the values of recovery varied from 85.73% to 98.01% for the six alkaloids. Thus, it suggested that the MRM mode of UPLC-QqQ-MS/MS have good reproducibility and precision.

**TABLE 2:** HPLC-QqQ-MS detection parameters, regression equations, linear range, precision, repeatability, and stability of six alkaloids.

Compound	t_R_ (min)	Regression equation	*R* ^2^	Linear range (ng·mL^-1^)	LOD (ng·mL^-1^)	LOQ (ng·mL^-1^)	Precision		Repeatability	Stability
							Intraday (n = 6)	Interday (n = 3)		
**31**	19.42	*y* = 0.0210*x* +0.0010	0.9997	1.11–355.84	0.04	0.11	1.11	1.60	2.71	1.03
**37**	36.35	*y* = 0.0012*x* −0.0002	0.9998	0.09–143.36	0.03	0.09	1.87	1.93	1.51	2.17
**39**	37.34	*y* = 0.0017*x* + 0.0013	0.9998	1.34–855.04	0.01	0.02	2.31	2.73	2.75	1.89
**33**	37.36	*y* = 0.0013*x* - 0.0010	0.9997	0.15–240.64	0.07	0.22	2.82	2.04	1.06	1.44
**42**	37.53	*y* = 0.0149*x* +0.0025	0.9998	0.12–38.4	0.12	0.41	1.03	1.71	2.26	2.09
**36**	38.14	*y* = 0.0231*x* −0.0035	0.9999	0.13–165.89	0.12	0.39	0.19	0.20	2.51	1.98

Note: In the regression equation, *x* is the ratio of peak area of analyte to peak area of internal standard reserpine, *y* the concentration of each analyte (ng·mL^-1^), and *R* ^2^ the correlation coefficient. LOD, limit of detection (S/N = 3), LOQ, limit of quantification (S/N = 10). Intra-, inter-day precision, repeatability, and stability are shown in RSD (%).

**TABLE 3:** Recoveries of the standard addition test of the six alkaloids.

Compound	80% spiking			100% spiking			120% spiking		
	Found (n = 3, mg)	Spiked	Recovery (%)	Found (n = 3, mg)	Spiked	Recovery (%)	Found (n = 3, mg)	Spiked	Recovery (%)
**31**	1.0875	0.88	93.15	1.0875	1.09	95.45	1.0875	1.32	96.74
**37**	0.0125	0.01	85.73	0.0125	0.012	88.11	0.0125	0.015	88.60
**39**	3.6125	2.91	96.66	3.6125	3.62	97.74	3.6125	4.35	98.01
**33**	0.0125	0.01	86.55	0.0125	0.012	88.32	0.0125	0.015	89.11
**42**	0.04	0.03	85.83	0.04	0.04	87.49	0.04	0.05	88.88
**36**	0.8025	0.65	90.60	0.8025	0.82	93.73	0.8025	0.97	95.29

## 3 Results

### 3.1 HPTLC-DESI-MSI analysis of main chemical components in different TBC processed products

**FIGURE 4:**
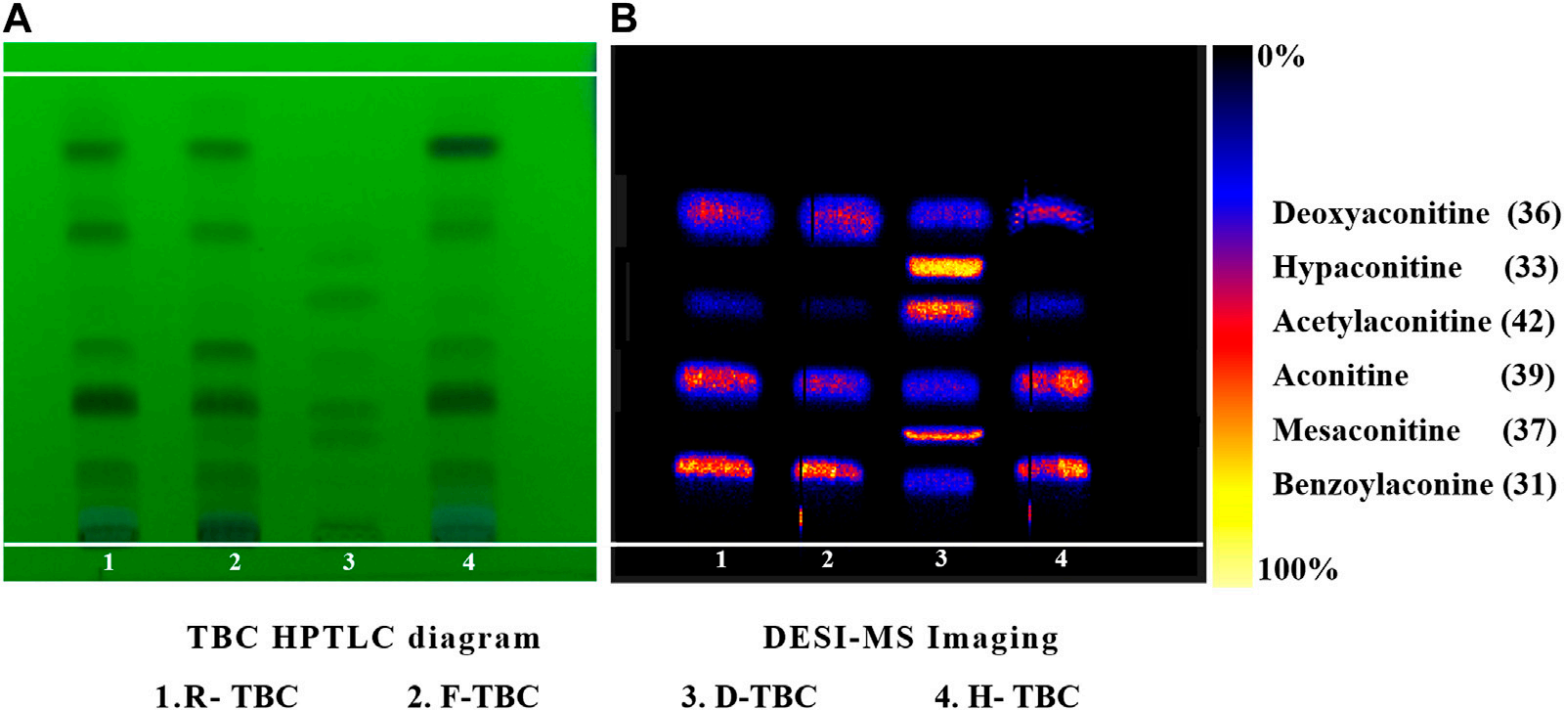
HPTLC and DESI diagrams of target control alkaloids in R-TBC, H-TBC, and F-TBC.

According to the investigation of the previous conditions and in alkaloid composition analysis, the response signal of the alkaloid components obtained using 70% methanol water +0.2% formic acid spray solvent was strong (Liu et al., 2022). Therefore, this condition was adopted in the present work. In literature, the contents of six components such as benzoylaconine (**31**), hypaconitine (**33**), deoxyaconitine (**36**), mesaconitine (**37**), aconitine (**39**), and acetylaconitine (**42**) in TBC were relatively high. The development conditions of TBC were investigated with the six components as the target (Supplementary Table S2). A relatively ideal result was obtained through optimization (Figure 4A). The HPTLC aluminum plate easily blackens under the action of alkaloid chromogenic agent, so the unfolding results were observed under UV 254 nm. Five obvious spots were found in different processed products of TBC, but these spots could not be completely compared with the mixed reference substance. In this regard, ambient mass spectrometry (MS) imaging was used for HPTLC direct *in situ* imaging and analysis. As shown in Figure 4B, the six components in the mixed control substance were well separated in HPTLC. The contents of hypaconitine (**33**) and mesaconitine (**37**) in the samples were lower than those of benzoylaconine (**31**), deoxyaconitine (**36**), aconitine (**39**), and acetylaconitine (**42**). Under limited conditions, the two components were not found in the imaging results. Based on the results of HPTLC and DESI-MSI, the main chemical constituents of TBC changed after processing HBW and FCS. The content of aconitine (**39**) in H-TBC was more than those of the raw product and F-TBC, while the contents of benzoylaconine (**31**), deoxyaconitine (**36**) and acetylaconitine (**42**) in H-TBC were less than R-TBC, but more than F-TBC. The results indicated that some chemical reactions possibly occurred during the processing of TBC.

### 3.2 HPLC-QqQ-MS for the determination of changes in the content of six alkaloids before and after TBC processing

The content of six alkaloids before and after TBC processing have been relative quantified using the signal intensity (peak area) of corresponding product ion by the MRM mode of HPLC-QqQ-MS/MS. The results showed that the content of aconitine (**39**) was up to 1 mg g^-1^ or higher and in H-TBC>R-TBC>F-TBC, benzoylaconitine (**31**), deoxyaconitine (**36**) and acetylaconitine (**42**) were all much lower than aconitine (**39**), but in R-TBC > H-TBC > F-TBC. The content of hypaconitine (**33**) and mesaconitine (**37**) was extremely low, only 0.005 mg g^-1^ in R-TBC and even lower after processing. It was found that the content of all six alkaloids decreased after processing by FCS, and all five alkaloids decreased except aconitine (**39**) increased after processing by HBW, which was consistent with the result Figure 4 of HPTLC-DESI-MSI assayed in 3.1. Due to the difference in the sensitivity of the detection methods, hypaconitine (**33**) and mesaconitine (**37**) were detected in very small amounts in HPLC-QqQ-MS/MS but not observed in HPTLC-DESI-MSI. In conclusion, the results of this content determination fully validated the feasibility of HPTLC-DESI-MSI to rapidly and accurately identify and visually present most of the variation differences of the components except for the very trace components.

### 3.3 Visual characterization of the chemical composition of different processed products of TBC by HPTLC-DESI-MSI

TLC can only analyze main components and needs a reference substance for comparison. The results have a high possibility of being false positive. DESI-MSI can characterize not only known components but also unknown components, and the *m/z* obtained can be used for identification (Mohana Kumara et al., 2019). Therefore, more accurate information can be obtained by HPTLC combined with DESI-MSI and can be directly visualized.

**FIGURE 5:**
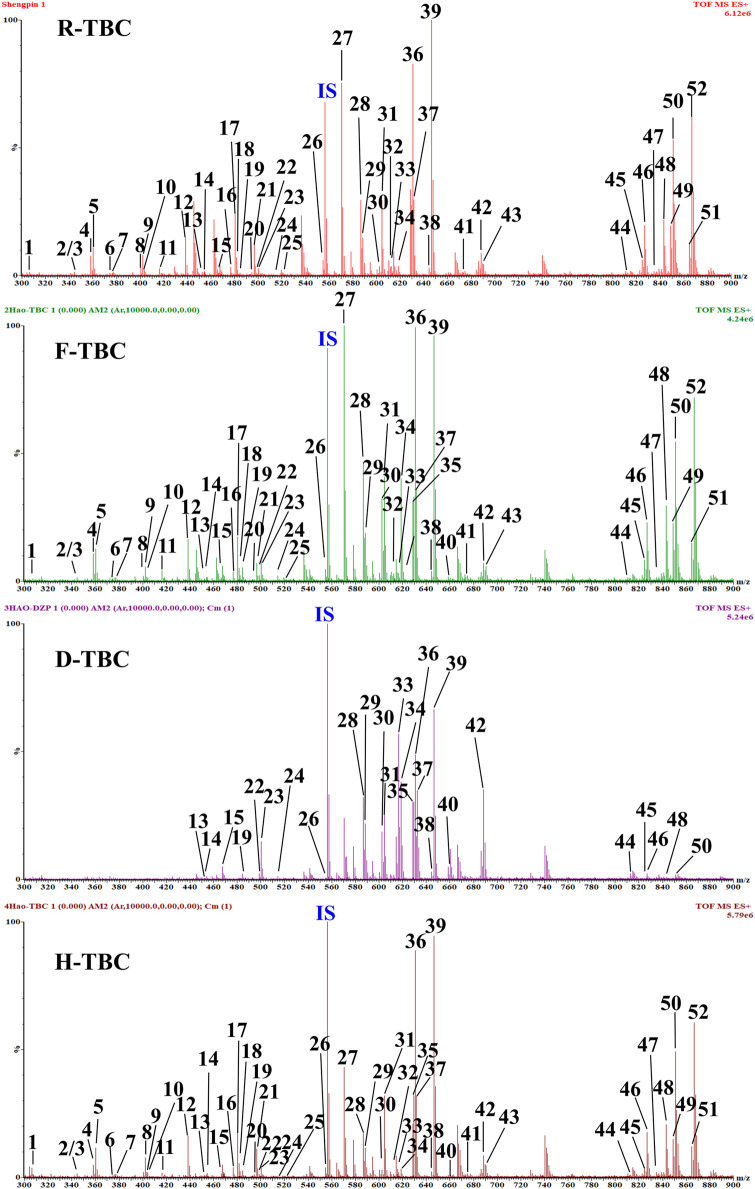
Signal of DESI-MSI acquired for mass range *m/z* 300–900 from HPTLC plate regions of the D-TBC, R-TBC, F-TBC and H-TBC in the positive ion mode. Putatively identfied alkaloids are labeled with *m/z* and compound number. See Table 4 for more details on the compound identification.

**FIGURE 6:**
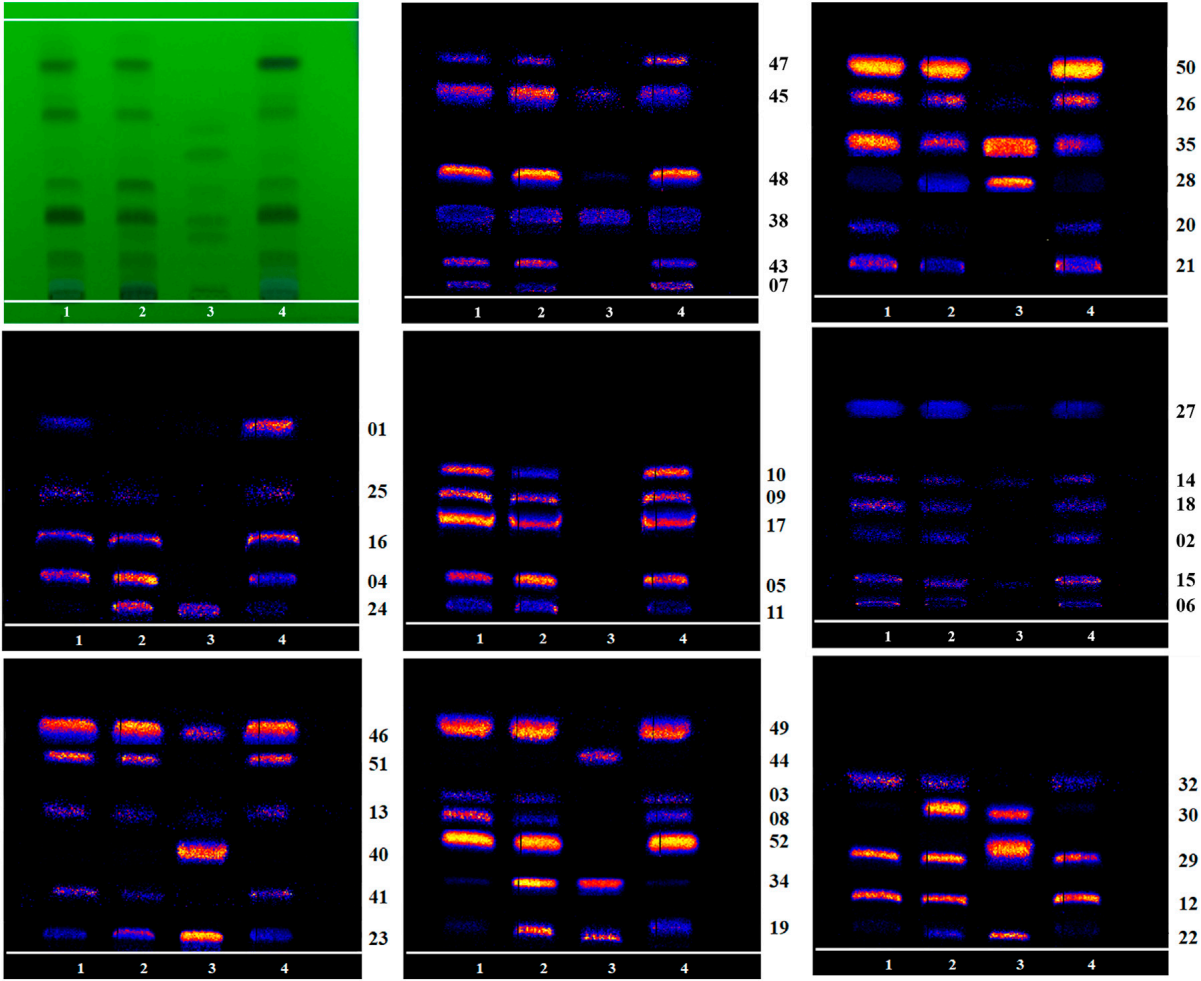
Mass Spectrometry Imaging comparison of 46 other identifiable alkaloid components on HPTLC (1: R-TBC, 2: F-TBC, 3: D-TBC, 4: H-TBC).

**FIGURE 7:**
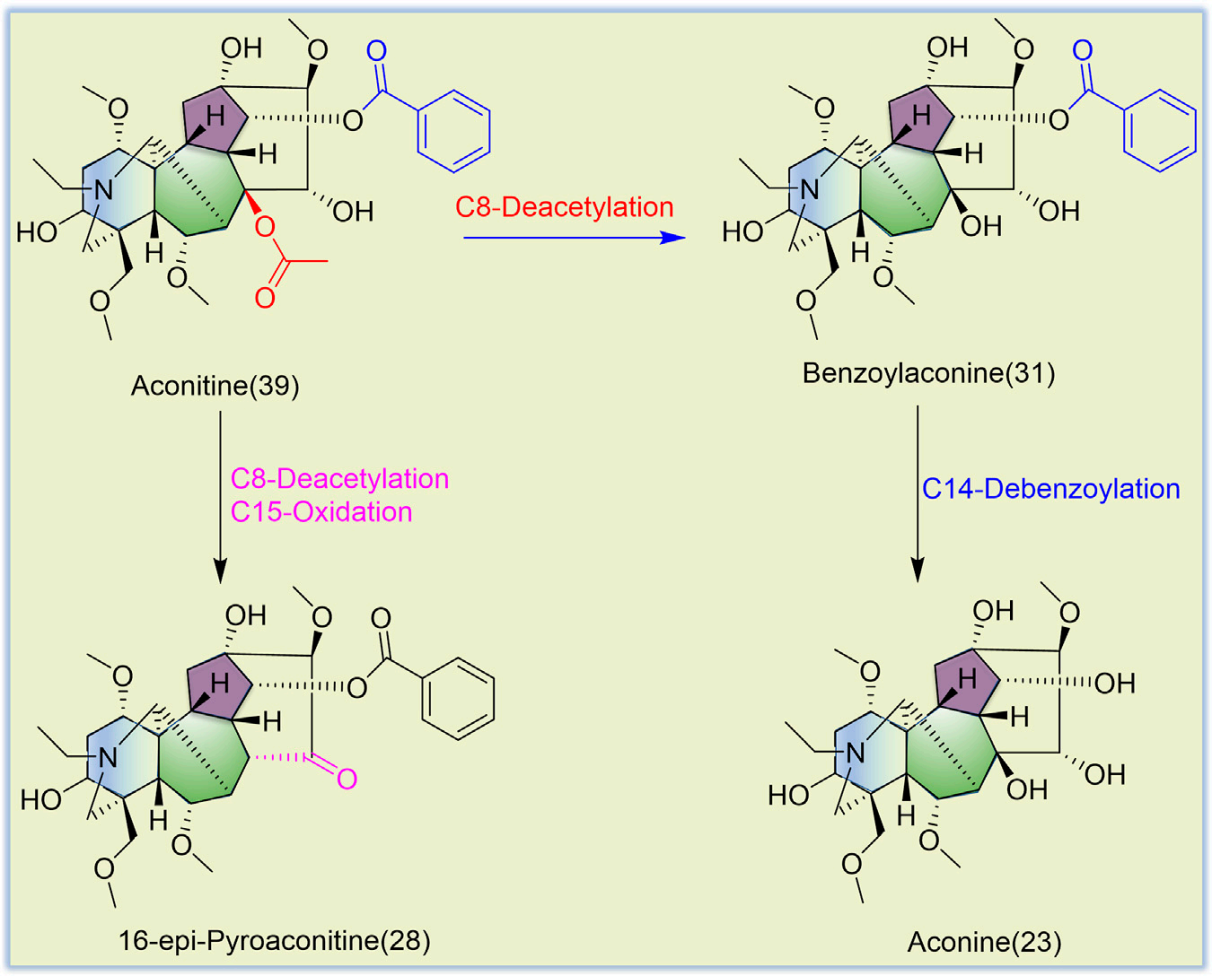
Possible mechanism of alkaloids transformation in the process of F-TBC.

TBC, as a plant of the genus *Aconitum*, contains a large number of alkaloids, such as diterpenoid and aliphatic alkaloids. Most of these alkaloids have *m/z* between 300 and 900. After the comparison with reference substances, accurate molecular weight, and related references and combined with the *m/z* of DESI-MSI, 52 chemical constituents were inferred and identified from TBC and its different processed products. The specific information is shown in Figures 5, 6 and Table 4. In Figure 5, most alkaloids are concentrated in the *m/z* 300–700 and *m/z* 800–900 region ranges. The aliphatic alkaloids *m/z* are mainly between 900–800 (Csupor et al., 2009), the contents of 8-palmitoleic acid −14-benzoylmesaconine (**46**), 14-benzoylaconine-8-palmitate (**48**), and 8-linolenic acid benzoyldeoxyaconine (**49**) are high in F-TBC. Combined with HPTLC and DESI-MSI, it is found 8-O-Linoleoyl-14-benzoylaconine **(52)** is less in F-TBC, while the content of lipodeoxyaconitine (**50**) is the largest in H-TBC. The *m/z* of diterpenoid alkaloids was mainly concentrated in the range of 500–700 (Wang et al., 2003), among which the alkaloids with the biggest difference were aconine (**23**), 16-epi-pyrodeoxyaconitine (**27**), deoxyaconitine (**36**), and aconitine (**39**). Compared with TBC raw products, the contents of aconitine (**39**) in F-TBC decreased significantly, while the contents of aconine (**23**) and 16-epi-pyrodeoxyaconitine (**27**) increased. In addition, the content of aconitine (**39**) in H-TBC was significantly increased. However, no significant change in 16-epi-pyrodeoxyaconitine (**27**) was found in H-TBC compared with that in R-TBC. Hence, the processing and detoxification mechanism of F-TBC may be related to the conversion mode of alkaloids shown in Figure 7, which is consistent with previous literature reports (Wang et al., 2010).

**TABLE 4:** List of possible compounds.

NO.	Compound	Formula	Theoretical Value	[M+H]	Observed Value	mDa	ppm	R_f_	Ref
01	Samandarin	C_19_H_31_NO_2_	305.2429	306.2433	306.2441	0.8	2.6	0.71	Habermehl and Ott (1976)
02	Bullatine A	C_22_H_33_NO_2_	343.2511	344.2590	344.2578	−1.2	−3.5	0.30	Yang et al. (2019)
03	Denudatine	C_22_H_33_NO_2_	343.2511	344.2590	344.2585	−0.5	−1.5	0.56	Reinecke et al. (1986)
04	Songorine	C_22_H_31_NO_3_	357.2304	358.2382	358.2376	−0.6	−1.7	0.17	Xu et al. (2019)
05	Napelline	C_22_H_33_NO_3_	359.2460	360.2539	360.2521	−1.8	−5.0	0.17	Yang et al. (2016)
06	Songorine N-​oxide	C_22_H_31_NO_4_	373.2460	374.2331	374.2323	−0.8	−2.1	0.06	Lu et al. (2010)
07	Karakanine	C_22_H_33_NO_4_	375.2410	376.2488	376.2478	−1.0	−2.7	0.03	Sultankhodzhaev (1993)
08	Dehydrolucidusculine	C_24_H_33_NO_4_	399.2448	400.2488	400.2475	−1.3	−3.2	0.47	Wada et al. (1985)
09	Acoridine	C_23_H_31_NO_5_	401.2216	402.2280	402.2268	−1.2	−3.0	0.46	Wang et al. (2016)
10	12-Acetyl-12-epi-napelline	C_24_H_35_NO_4_	401.2566	402.2644	402.2638	−0.6	−1.5	0.56	Lu et al. (2010)
11	Guan Fu base Z	C_24_H_33_NO_5_	415.2359	416.2437	416.2429	−0.8	−1.9	0.07	Reinecke et al. (1986)
12	Neoline	C_24_H_39_NO_6_	437.2777	438.2856	438.2836	−2.0	−4.6	0.20	Wei et al. (2019)
13	Dehydrodelcosine	C_24_H_37_NO_7_	451.2627	452.2648	452.2631	−1.7	−3.8	0.49	Takayama et al. (1988)
14	Chasmanine	C_25_H_41_NO_6_	451.6124	452.3012	452.2997	−1.5	−3.3	0.50	Wei et al. (2019)
15	14-O-acetylsenbusine A	C_25_H_39_NO_7_	465.2727	466.2805	466.2787	−1.5	−3.2	0.16	Frejat et al. (2017)
16	Liangshantine/Diacetylheteratisine	C_26_H_37_NO_7_	475.2570	476.2648	476.2633	−1.5	−3.1	0.32	Peiqin et al. (1997)
17	14-Acetylneoline	C_26_H_41_NO_7_	479.2883	480.2961	480.2949	−1.1	−2.3	0.37	Díaz et al. (2000)
18	1-O-benzoylkaracoline	C_29_H_39_NO_5_	481.2676	482.2906	482.2894	−1.2	−2.5	0.38	Xu et al. (2016)
19	Pseudaconine	C_25_H_41_NO_8_	483.2832	484.2910	484.2893	−1.7	−3.5	0.08	Tang et al. (2014)
20	Olividine	C_26_H_39_NO_8_	493.2664	494.2744	494.2754	−1.0	−2.0	0.23	Grandez et al. (2002)
21	8-Acetyl-15-hydroxyneoline	C_26_H_41_NO_8_	495.2832	496.2910	496.2900	−1.0	−2.0	0.11	Wang et al. (2010)
22	10-Hydroxyllycoctonine	C_26_H_43_NO_8_	497.2987	498.3067	498.3052	−1.5	−3.0	0.07	Zhang and Jia (1999)
23	Aconine	C_25_H_41_NO_9_	499.2781	500.2860	500.2844	−1.6	−3.2	0.06	Chen S et al. (2014)
24	8-O-methylaconine	C_26_H_43_NO_9_	513.2938	514.3016	514.3010	−0.6	−1.2	0.06	Song et al. (2019)
25	1,14-Diacetylneoline	C_28_H_43_NO_8_	521.2926	522.3067	522.3050	−1.7	−3.3	0.46	Fuente et al. (1988)
26	Kongboendine	C_32_H_43_NO_7_	553.3040	554.3118	554.3118	−0.0	−0.0	0.68	A et al. (2002)
27	16-epi-Pyrodeoxyaconitine	C_32_H_43_NO_8_	569.2989	570.3067	570.3068	−0.1	−0.2	0.77	Wang et al. (2011)
28	16-epi-Pyroaconitine	C_32_H_43_NO_9_	585.2938	586.3019	586.3016	0.3	0.5	0.41	Wang et al. (2011)
29	Benzoyldeoxyaconine	C_32_H_45_NO_9_	587.3094	588.3173	588.3156	−1.3	−2.2	0.33	Lu et al. (2010)
30	Austroconitine B/Geniculatine B	C_33_H_47_NO_9_	601.3251	602.3329	602.3301	−2.8	−4.6	0.50	Liu et al. (2022)
31	Benzoylaconine	C_32_H_45_NO_10_	603.3043	604.3122	604.3132	1.0	1.7	0.16	#
32	13-Deoxyanhydroaconitine	C_34_H_45_NO_9_	611.3036	612.3173	612.3154	−1.9	−3.1	0.59	Li et al. (2003)
33	Hypaconitine	C_33_H_45_NO_10_	615.3043	616.3122	616.3124	0.2	0.3	0.59	#
34	8-​O-​methyl-​14-benzoylaconine	C_33_H_47_NO_10_	617.3256	618.3278	618.3267	−1.1	−1.8	0.23	Li et al. (2018)
35	1-Demethoxyyunaconitinone/Anhydroaconitine	C_34_H_45_NO_10_	627.3043	628.3122	628.3134	1.2	1.9	0.56	Wang et al. (2017)
36	Deoxyaconitine	C_34_H_47_NO_10_	629.3200	630.3278	630.3278	0.0	0.0	0.67	#
37	Mesaconitine	C_33_H_45_NO_11_	631.2993	632.3071	632.3081	1.0	1.6	0.22	#
38	(−) -(a-c)-8β-Acetoxy-14α-benzoyloxy-N-ethyl-13β,15α-dihydroxy-lα, 6α,l6β,18-tetramethoxy-19-oxo-aconitane	C_34_H_45_NO_11_	643.2993	644.3071	644.3053	−1.8	−2.8	0.28	Wei et al. (2019)
39	Aconitine	C_34_H_47_NO_11_	645.3149	646.3227	646.3249	2.2	3.4	0.33	#
40	Yunaconitine	C_35_H_49_NO_11_	659.3326	660.3384	660.3372	−1.2	−1.8	0.36	Sultankhodzhaev et al. (1980)
41	Polyschistine A	C_36_H_51_NO_11_	673.3462	674.3540	674.3527	−1.3	−1.9	0.20	Wang et al. (1985)
42	3-​Acetylaconitine	C_36_H_49_NO_12_	687.3255	688.3333	688.3345	1.2	1.7	0.48	#
43	Nagaconitine A	C_36_H_51_NO_12_	689.3411	690.3490	690.3505	1.5	2.2	0.11	Zhao et al. (2017)
44	8-​Palmitoyl-benzoylhypa​-conine	C_47_H_73_NO_10_	811.5298	812.5313	812.5346	3.3	4.1	0.68	Kitagawa et al. (1984)
45	8-Heptadecenoic acid-benzoylhypa​conine	C_48_H_73_NO_10_	823.5202	824.5313	824.5331	1.8	2.2	0.71	Kitagawa et al. (1984)
46	8-Palmitoleic acid −14-benzoylmesaconine	C_48_H_75_NO_10_	825.5489	826.5469	826.5487	1.8	2.2	0.79	Kitagawa et al. (1984)
47	8-Linolenicacid-benzoylhypaconine	C_50_H_75_NO_9_	833.5513	834.5520	834.5515	−0.5	−0.6	0.83	Kitagawa et al. (1984)
48	14-Benzoylaconine-8-palmitate	C_48_H_75_NO_11_	841.5340	842.5418	842.5410	−0.8	−0.9	0.43	Kitagawa et al. (1984)
49	8- Linolenic acid -benzoyldeoxyaconine	C_50_H_73_NO_10_	847.5326	848.5313	848.5316	0.3	0.4	0.78	Kitagawa et al. (1984)
50	Lipodeoxyaconitine	C_50_H_75_NO_10_	849.5391	850.5469	850.5478	0.9	1.1	0.80	Kitagawa et al. (1984)
51	8-Linolenicacid-14-benzoylaconine	C_50_H_73_NO_11_	863.5234	864.5262	864.5284	2.2	2.5	0.67	Kitagawa et al. (1984)
52	8-O-Linoleoyl-14-benzoylaconine	C_50_H_75_NO_11_	865.5340	866.5418	866.5427	0.9	1.0	0.39	Kitagawa et al. (1984)

As shown in Figure 5, compared with R-TBC, the relative contents of napelline (**5**), pseudaconine (**19**), 8-O-methylaconine (**24**), 8-linolenicacid-lenzoylhypaconine (**47**) in T-TBC and H-TBC increased significantly, while the contents of dehydrolucidusculine **(8)**, 14-acetylneoline (**17**), 8-acetyl-15-hydroxyneoline (**21**), kongboendine (**26**), and 1-demethoxyyunaconitinone/anhydroaconitine (**35**) decreased significantly. Interestingly, songorine (**4**), nagaconitine A (**43**) and 8-heptadecenoic acid-benzoylhypaconine (**45**) increased in F-TBC but decreased in H-TBC, samandarin (**1**) and karakanine (**7**) decreased in F-TBC and increased in H-TBC. In addition, it was worth noting that some components increased irregularly. For example, compared with R-TBC, the content of 10-hydroxyllycoctonine (**22**), 16-epi-pyroaconitine (**28**), austroconitine B/geniculatine B **(30)** and 8-O-methyl-14-benzoylaconine (**34**) in F-TBC increased, while the content of acoridine (**9**), 12-acetyl-12-epi-napelline (**10**), olividine (**20**), and 1, 14-diacetylneoline (**25**) decreased, unchanged in H-TBC. Finally, compared with R-TBC, the contents of guan fu base Z (**11**) and 16-epi-pyrodeoxyaconitine (**27**) in H-TBC decreased, but the two components did not change in F-TBC. Based on the changes in these chemical components, the processing mechanism of F-TBC and H-TBC is very different and worthy of further study.

## 4 Discussion

In this study, the combined technique of HPTLC and DESI-MSI was adopted, instead of the commonly used HPLC and LC-MS detection, because this combined technique requires simple equipment operation, no complicated sample preparation process, fast analysis time and less solvent consumption, which is a more time-saving and environment-friendly analysis method (Tang et al., 2021; Milojković-Opsenica et al., 2022). In addition, DESI-MSI can be used for *in situ* detection and direct identification of compounds according to ion mass-charge ratio, which makes up for the limitation of HPTLC in identifying compounds dependent on reference substances (Parrot et al., 2018; Ristivojevi´c et al., 2020). The results showed that HPTLC was used for chromatographic separation of extracts, and only 5 main compound spots could be seen under fluorescence, and 52 compounds could be identified by *in situ* detection combined with DESI-MSI, and the relative content difference between the extracts before and after processing with TBC was visually displayed. The content of the six target alkaloids before and after different processing methods were also detected by HPLC-QqQ-MS, and the differences in content changes were highly consistent with the results of HPTLC-DESI-MSI analysis. This study further confirms that HPTLC-DESI-MSI is a simple, rapid, efficient, and solvent-saving technique with significant advantages in the separation and characterization of chemical components in complex samples.

The results showed certain changes in alkaloids after the processing of F-TBC and H-TBC, such as pseudaconine (**19**), 8-O-methylaconine (**24**), and deoxyaconitine (**36**) were decreased. However, the changes of compounds caused by the two processing methods were not identical. Compared with aconitine (**39**) increased in H-TBC, but in F-TBC was decreased more, and aconine (**23**), 16-epi-pyrodeoxyaconitine (**27**), 16-epi-pyroaconitine (**28**), benzoylaconine (**31**), 8-O-methyl-14-benzoylaconine (**34**), 8-heptadecenoic acid-benzoylhypaconine (**45**), 14-benzoylaconine-8-palmitate (**48**), 8-O-linoleoyl-14-benzoylaconine (**52**), and other components increased. Almost all of these added components were the products of degradation or hydrolysis of ester aconitine and aconitine. Studies have been confirmed that the main toxic components of TBC are diester-diterpenoid alkaloids such as aconitine, deoxyaconitine and 3-acetylaconitine (Singhuber et al., 2009; Zhang et al., 2014). FCS mainly contains tannin and phenolic acids. In the processing process, FCS can accelerate the leaching of alkaloids and reduce the concentration of toxic alkaloids (Li et al., 2021; Zha et al., 2000). At the same time, some of the diester-diterpenoid alkaloids were converted into less toxic monoester-diterpenoid or non-esterified diterpene alkaloids (Chen R et al., 2014) (Figure 6). However, compared with F-TBC, aconitine (**39**) increased, aconine (**23**), 16-epi-pyrodeoxyaconitine (**27**), and benzoylaconine (**31**) almost unchanged, while samandarin (**1**), 14-O-acetylsenbusine A (**15**), and 8-linolenicacid-benzoylhypaconine (**47**) increased slightly. Songorine (**4**), Guan Fu base Z (**11**), 8-acetyl-15-hydroxyneoline (**21**), 13-deoxyanhydroaconitine (**32**), nagaconitine A (**43**), and 8-Heptadecenoic acid-benzoylhypaconine (**45**) decreased slightly, but the changes of these compounds did not show obvious transformation trend, and their chemical mechanism was unclear. These results also indicated that the processing mechanisms of F-TBC and H-TBC were completely different.

According to the processing experiment, H-TBC and F-TBC belong to the non-heating processing methods of traditional Tibetan medicine, which are obviously different from other heating processing methods such as boiling, steaming and frying. It has been reported that heating processing method could hydrolyze or pyrolyze diester-diterpenoid toxic alkaloids, such as deoxyaconitine (**36**), aconitine (**39**), 3-acetylaconitine (**42**) into monoester-diterpenoid or non-esterified diterpene alkaloids (Wang et al., 2010). For example, the content of aconitine (**39**) was less than one-tenth the amount of raw aconitine after heating and was undetectable (Zhi et al., 2020). Although the content of deoxyaconitine (36) and 3-acetylaconitine (42) was decreased by non-heating processing of H-TBC and F-TBC not as significant as that of heating processing, especially, the content of aconitine (39) was instead increased during the HBW processing. Previous studies proved the toxicity after heating type processing of TBC < F-TBC<H-TBC, and the efficacy was the drug effect was consistent with the toxicity, which indicated that the diester-diterpenoid alkaloids were not only the toxic substances of TBC, but also the key pharmacodynamic substances (Zhou et al., 2015).

In conclusion, the chemical transformation mechanism of TBC non-heating processing method was significantly different from that of heating processing method. Compared with the heating processing method, the chemical transformation mechanism of the non-heating processing was more complex and diverse, and the main chemical transformation mechanism of the processing of F-TBC was basically clear through this study. While the chemical composition change of H-TBC was not found out obvious rules. Therefore, the mechanism of the two non-heating processing methods needs to be further studied, and the principle of detoxification needs to be further clarified through body experiments, which will provide guidance for the correct clinical use of the traditional non-heating processing F-TBC and H-TBC, and expanding the application range of TBC.

## 5 Conclusion

In this study, a combination of HPTLC and DESI-MSI was developed for rapid and high-resolution characterization of alkaloid changes in raw and processed products of TBC. A total of 52 chemical constituents were identified in TBC, and 27 chemical constituents were changed during processing, including 9 common constituents in F-TBC and H-TBC, 12 chemical constituents unique in F-TBC, and 6 constituents unique in H-TBC. According to the changes in diester and monoester alkaloids, these components of F-TBC and H-TBC did not change much, and a large number of diester alkaloids were retained. Monoester and ethanolamine alkaloids increased or decreased. From the change of the whole chemical composition, F-TBC has a certain change rule. FCS contains a large amount of tannins and is acidic, which can accelerate the hydrolysis of toxic diester alkaloids. Tannins in FCS can complex with alkaloids to form insoluble substances. H-TBC does not have this rule. In conclusion, HPTLC-DESI-MSI is a feasible method to quickly and accurately identify and visualize most of the variation differences in components except for very trace components, which can be used to separate and identify alkaloids of different processed varieties of TBC. This study not only provides an alternative method for the traditional separation and identification of secondary metabolism but also develops a method reference for the study of processing mechanism and quality control of ethnic medicine.

## Data Availability

The original contributions presented in the study are included in the article/Supplementary Material, further inquiries can be directed to the corresponding authors.
