# Supplementary material for: Rapid visual characterization of alkaloid changes in traditional processing of Tibetan medicine Aconitum pendulum by high-performance thin-layer chromatography coupled with desorption electrospray ionization mass spectrometry imaging
Source: Front Pharmacol. 2023 Apr 21;14:1104473. doi: 10.3389/fphar.2023.1104473 (PMC10160446; doi:10.3389/fphar.2023.1104473)
Supplement: Supplementary file 1 [file Table1.DOCX]

Supplementary Material

**Table S1.** Results of HPLC-QqQ-MS for the determination of six alkaloids in R-TBC, F-TBC and H-TBC (n=3).

| **Compound content (mg. g^-1^)** | **R-TBC** | **F-TBC** | **H-TBC** |
| --- | --- | --- | --- |
|  |  |  |  |
| **31** | 0.435 | 0.399 | 0.407 |
| **37** | 0.005 | 0.004 | 0.004 |
| **39** | 1.445 | 1.043 | 1.504 |
| **33** | 0.005 | 0.004 | 0.004 |
| **42** | 0.016 | 0.011 | 0.015 |
| **36** | 0.321 | 0.279 | 0.293 |
