# Supplementary material for: Rapid visual characterization of alkaloid changes in traditional processing of Tibetan medicine Aconitum pendulum by high-performance thin-layer chromatography coupled with desorption electrospray ionization mass spectrometry imaging
Source: Front Pharmacol. 2023 Apr 21;14:1104473. doi: 10.3389/fphar.2023.1104473 (PMC10160446; doi:10.3389/fphar.2023.1104473)
Supplement: Supplementary file 2 [file Table2.DOCX]

Supplementary Material

**Table S2. Mass spectrum information of reference substance**

| **Compound** | **Formula** | **the Theoretical Value** | **[M+H]** | **the Observed Value** | **mDa** | **ppm** |
| --- | --- | --- | --- | --- | --- | --- |
| Benzoylaconine | C_32_H_45_NO_10_ | 603.3043 | 604.3122 | 604.3132 | 1.0 | 1.7 |
| Mesaconitine | C_33_H_45_NO_11_ | 631.2993 | 632.3071 | 632.3081 | 1.0 | 1.6 |
| Aconitine | C_34_H_47_NO_11_ | 645.3149 | 646.3227 | 646.3249 | 2.2 | 3.4 |
| Acetylaconitine | C_36_H_49_NO_12_ | 687.3255 | 688.3333 | 688.3345 | 1.2 | 1.7 |
| Hypaconitine | C_33_H_45_NO_10_ | 615.3043 | 616.3122 | 616.3124 | 0.2 | 0.3 |
| Deoxyaconitine | C_34_H_47_NO_10_ | 629.3200 | 630.3278 | 630.3278 | 0.0 | 0.0 |
